# Bochdalek hernia in pregnancy complicated with eclampsia: a case report

**DOI:** 10.1515/med-2026-1456

**Published:** 2026-06-08

**Authors:** Gordana Vukcevic, Simonida Kavaric

**Affiliations:** Clinic for Gynecology and Obstetrics, Clinical Center of Montenegro, Podgorica, Montenegro

**Keywords:** Bochdalek hernia, eclampsia, pregnancy, posterior reversible encephalopathy

## Abstract

**Objectives:**

Asympthomatic Bochdalek hernia (BH) is extremely rare in pregnant women. Here, we present a 23-year-old pregnant woman admitted to the hospital with a first pregnancy at 38 gestational weeks and a medical history of hypertensive disorders.

**Case presentation:**

During hospitalization, she developed an eclamptic attack, and an emergency cesarean section was performed. After the operation, the patient was admitted to the intensive care unit (ICU) because of her poor general condition. At the ICU, she was subjected to multidisciplinary treatment. During the diagnostic protocols, a prolapse of the abdominal organs into the left chest cavity was noted, with atelectasis of the left lung and contralateral compression of the mediastinum. The thoracic surgeon performed the thoracoscopic surgical procedure. During the postoperative course, the patient was cardiorespiratory stable. However, due to neurological problems, the patient was transferred to the Department of Neurology where the diagnosis was made – syndrome of posterior reversible encephalopathy (PRES).

**Conclusions:**

Although maternal Bochdalek hernias are uncommon, timely diagnosis and coordinated multidisciplinary management are critical to optimizing both maternal and fetal health.

## Introduction

Congenital diaphragmatic hernia (CDH) is a disorder in which the diaphragm develops abnormally, causing the contents of the abdomen to protrude into the thoracic cavity [1]. Although CDH is typically diagnosed during pregnancy or the first few months after delivery, 5–25 % of cases may be discovered later, during regular exams, or as a result of gastrointestinal or respiratory issues [1], 2]. Different forms of hernias can be distinguished on the basis of where the diaphragm defect is located. Bochdalek hernias (BH) are the most common type of hernia (70–75 %) and are caused by a defect in the posterolateral region of the diaphragm [2]. This oddity was initially reported by Bochdalek in 1848, and in 80–90 % of cases, it occurs on the left side [2]. Respiratory distress syndrome after delivery and pulmonary hypoplasia on the afflicted side are linked to larger BH abnormalities [3]. Small abnormalities can be asymptomatic until the contents of the abdomen herniate into the thoracic cavity, which can have respiratory repercussions. They are not linked to a deficiency in lung development. The intra-abdominal organ that most frequently migrates through diaphragmatic defects is the colon, which can clog the large intestine [3].

Reduction of the abdominal contents and repair of the defect by thoracotomy or laparotomy are the main parts of the management of a BH [4]. Laparotomy has historically been utilized for surgical repair; however, with the advent of laparoscopy, laparoscopic treatment has become more popular since it requires fewer hospital stays and allows for an earlier start time [4]. There have been reports of both thoracoscopic and laparoscopic BH repairs [4]. Larger flaws may lead to a decrease in intra-abdominal contents, although smaller flaws are simpler to fix [4].

BH primarily manifests in children, affecting approximately 1 in 2,200–12,500 live births [5]. However, the presentation of a Bochdalek hernia in an adult is exceptionally rare. Gastric volvulus and strangulation are the most common complications of BH in adults and require immediate emergency surgery [4], [5], [6]. More interestingly, in the adult population, the detection of BH in pregnant women has almost never been reported in the literature.

Therefore, in the present study, we report a young pregnant woman with preeclampsia and eclampsia who underwent surgery after childbirth for BH and who was followed up in our clinic. Data from this report could be highly valuable for clinical practice, especially for gynecologists and obstetricians.

## Case presentation

### Ethical approval

This study was conducted in accordance with the ethical principles outlined in the Declaration of Helsinki (as revised in 2013). It has been reviewed and approved by the Ethical committee of the Clinical Centre of Montenegro (Approval No. 03/01–24020/1).

### Informed consent

Informed consent was obtained from the patient.

A 23-year-old woman in her first pregnancy was admitted to the Clinic for Gynecology and Obstetrics due to high blood pressure and loss of consciousness. The patient was shaking but without foaming at the mouth or biting her tongue, without nausea, dizziness or vomiting. Clinical, laboratory and ultrasound examinations were carried out after admission. Anemia, elevated values ​​of liver enzymes (aspartate aminotransferase (AST), alanine transaminase (ALT), alkaline phosphatase (ALP), and lactate dehydrogenase (LDH)) and D-dimer are noted in the laboratory findings. During the ultrasound examination, fetal biometry was found to be 36 gestation weeks, with normal amniotic fluid and normal flow through the umbilical blood vessels. During the course of hospitalization, the patient experienced an eclamptic attack, after which the pregnancy was terminated by an emergency cesarean section, and a live male child was born (body weight 2,790 g, Apgar score 8/9).

**Figure 1:**
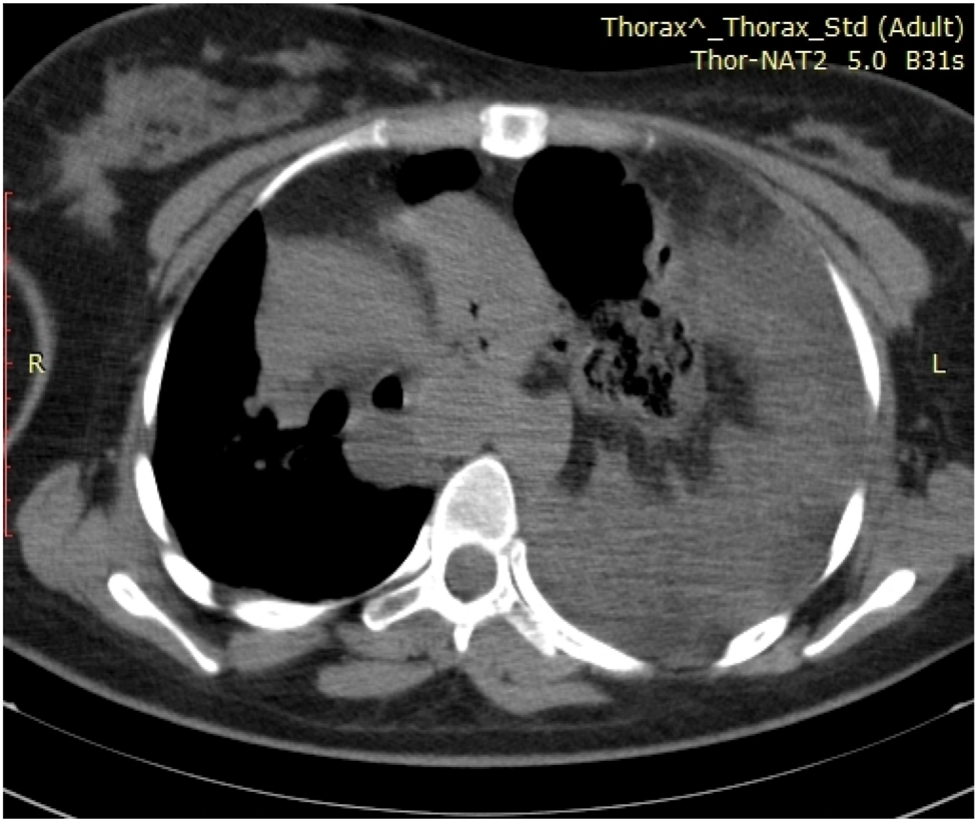
Image of a chest CT scan.

**Figure 2:**
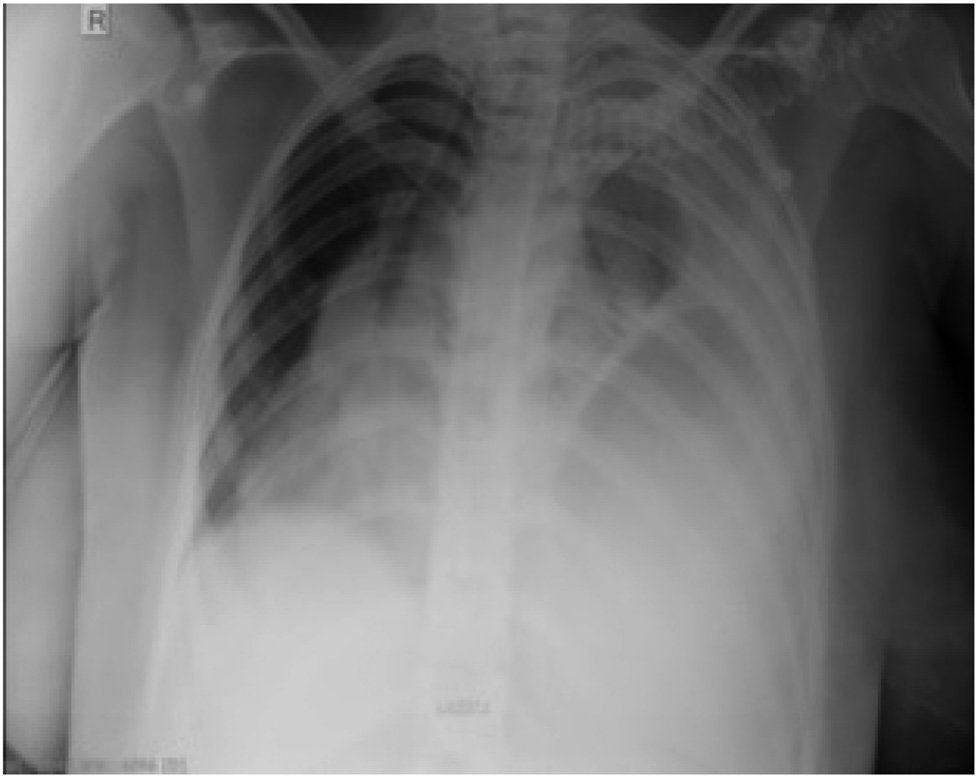
Image of a chest X-ray.

After cesarean section, the patient was admitted to the intensive care unit. In the waking phase, she breathed spontaneously with oxygen supplementation through a mask, but she was hypertensive and tachycardic. In the following days, the patient was conscious, communicative, hypertensive, prescribed therapy and dependent on oxygen. CT of the chest and CT of the endocranium were performed. Chest CT verified the existence of diaphragmatic hernia on the left side with prolapse of the hollow abdominal organs in the left hemisphere, atelectasis of the left lung wing and compression/displacement of the mediastinum contralaterally (Figure 1). The right lung parenchyma showed reduced aeration with multiple pulmonary consolidations (Figure 2). Owing to worsening of the patient’s general condition, on the 4th day after admission to the intensive care unit, the chest surgeon performed a thoracosurgical intervention, i.e., left subcostal laparatomia with repositioning of the abdominal organs.

**Figure 3:**
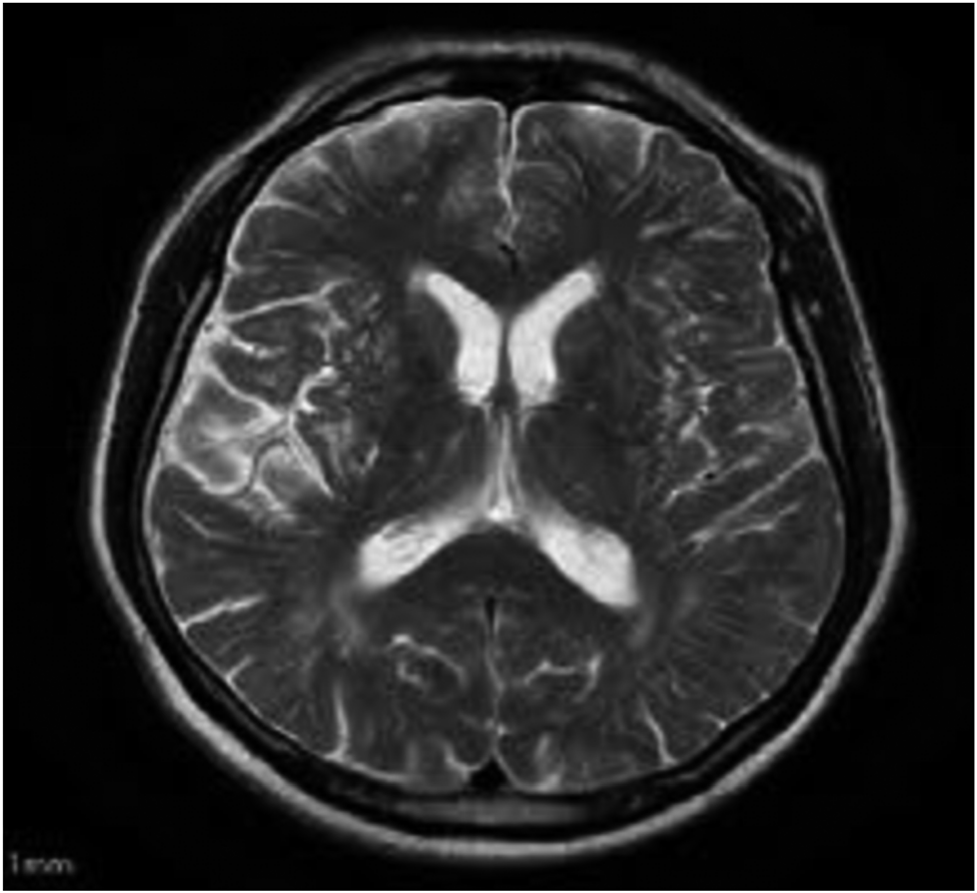
Magnetic resonance imaging (MRI) of the endocranium.

During further follow-up, the patient was cardiocirculatory, stabilized with mechanical support, and afebrile with appropriate diuresis. After a few days, she was separated from mechanical ventilation, extubated, conscious, poorly communicative, occasionally agitated and ordered without neurological lateralization. On several occasions, X-ray and CT of the abdomen, chest, and endocranium as well as MRI of the endocranium and MSCT of blood vessels of the head and neck were performed. Furthermore, owing to bilateral pleural effusion, thoracentesis was performed on the left side, and approximately 750 mL of fluid was evacuated. On the day of discharge from the intensive care unit, the patient was awake, did not communicate, occasionally confabulated, and periodically executed orders without neurological lateralization. The patient was then transferred to the Clinic of Neurology for further treatment. MR examination of the endocranium verified bilateral parietal and left frontal zones of altered signals, mainly cortical and subcortical with gyral signals, and detected signs of hypersignal corresponding to minor hemorrhagic transformation, which is in accordance with the clinical picture corresponding to syndrome of posterior reversible encephalopathy (PRES) (Figure 3).

## Discussion

In this case report, we present a unique occurrence of BH detected during the treatment of preeclamptic complications during pregnancy. Owing to this complication, the pregnant woman was admitted to the Clinic for Gynecology and Obstetrics, where she immediately experienced an eclamptic attack. Therefore, an emergency cesarean section was performed, and a live male child was born. During subsequent diagnostic procedures, a BH was accidentally discovered and surgically repaired. Although asymptomatic in this pregnant woman, a BH during pregnancy certainly poses a risk to the course of pregnancy and childbirth, especially considering that this woman suffers from preeclampsia [7], 8].

There are several contributing reasons for this case report. First, the presence of asymptomatic BH in adults is extremely rare, and to date, approximately 150 cases of occult asymptomatic BH in adults have been reported in the literature [9], 10]. More interestingly, the occurrence of BH during pregnancy is almost undocumented, especially in patients with preeclampsia.

Although, preeclampsia most commonly manifests after 5 months of pregnancy, in rare instances it can present prior to this period. Generally, preeclampsia and related conditions in pregnancy remain major contributors to maternal and perinatal morbidity and mortality [11]. Symptoms of preeclampsia may include dyspnea, tachycardia, altered mental status, heightened anxiety, and others [11], 12].

On the other hand, Bochdalek hernias are posterior diaphragmatic defects resulting from the incomplete closure of the pleuroperitoneal membrane during fetal development. This anomaly allows retroperitoneal structures, such as retroperitoneal fat or the left kidney, to herniate through defect [5], 6]. CDH, most commonly identified during infancy, are traditionally reported to occur more frequently on the left side, likely as a result of protective positioning of the liver on the right. However, this predominance has been recently challenged, particularly in adult populations. The delayed closure of the left posterior diaphragmatic foramen during fetal development can be another reason for mentioned asymmetry. Pulmonary complications are the most common [5], 6].

These hernias may be also detected in patients undergoing abdominal computed tomography angiography (CTA). In adults these hernias are usually right-sided and observed more frequently in female patients. Most of these hernias are small, with abdominal organ herniation occurring in up to 30 % of cases. Among individuals who survive CDH, half experience good long-term outcomes, remaining asymptomatic and without functional limitations [6]. It seems, that the majority of adult patients remain asymptomatic and do not require treatment. Symptomatic cases are typically diagnosed early in life, with an increasing number identified prenatally due to advancements in prenatal imaging and ongoing fetal surveillance [13]. Diligent screening and delivery at a tertiary care facility in order to provide specialized support are crucial. Given the identification of multiple comorbidities, long-term follow-up is imperative as the population of survivors increases. Accordingly, patient education should encompass not only the symptoms related to Bochdalek hernia and its surgical treatment but also potential long-term complications [4], 13].

Our patient also had asymptomatic BH without any health problems until 36 weeks of gestation and was diagnosed with preeclampsia and eclamptic attack. The complication of BH during pregnancy, such as hernia strangulation with consequent gangrene of the entire stomach, was recorded very recently by other authors as a life-threatening complication for both the mother and the fetus [14]. In addition, the onset of symptoms in previously asymptomatic pregnant women with congenital BH can be found in different literature sources [15], 16]. However, whether BH and preeclampsia are associated and potentiate each other is still unknown to the best of our knowledge. Therefore, the present information could be of great clinical importance in terms of increasing attention given that pregnancy-related BH may be complicated with preeclampsia and eclampsia.

Furthermore, posterior reversible encephalopathy (PRES) is frequently observed in clinical neurological practice as a complication of severe preeclampsia and eclampsia [17]. Various conditions, such as hypertension, preeclampsia, eclampsia, toxic agents, autoimmune diseases, and epileptic seizures, are present. The characteristic symptoms are nausea, vomiting and focal neurological signs. The main pathophysiological mechanism leading to the development of this syndrome is cerebral vasogenic edema, which is a consequence of abnormal blood flow through the brain. MRI and CT are used to diagnose the syndrome. PRES is a reversible syndrome, but in a small number of patients, neurological deficits remain permanent. Additionally, up to 15 % of cases die due to hemorrhage [18], 19]. In the present case report, PRES was diagnosed after delivery, and the woman was detained at the neurology clinic, which gives this case additional practical importance.

## Conclusions

The present study presents a unique case of BH during pregnancy complicated with eclampsia, PRES and delivery. Despite the rarity of maternal Bochdalek hernias during pregnancy, early diagnosis and appropriate treatment via multidisciplinary care are essential for maternal and fetal outcomes. The data presented here may therefore be of great clinical importance and may help clinicians first be familiar with this situation and then make the best decisions regarding the care of these pregnant women, diagnosis and therapy.
